# Supplementary material for: ORFeome Phage Display Reveals a Major Immunogenic Epitope on the S2 Subdomain of SARS-CoV-2 Spike Protein
Source: Viruses. 2022 Jun 17;14(6):1326. doi: 10.3390/v14061326 (PMC9229677; doi:10.3390/v14061326)
Supplement: Supplementary file 1 [file viruses-14-01326-s001.zip › viruses-1757271-supplementary.pdf]

Supplementary Material

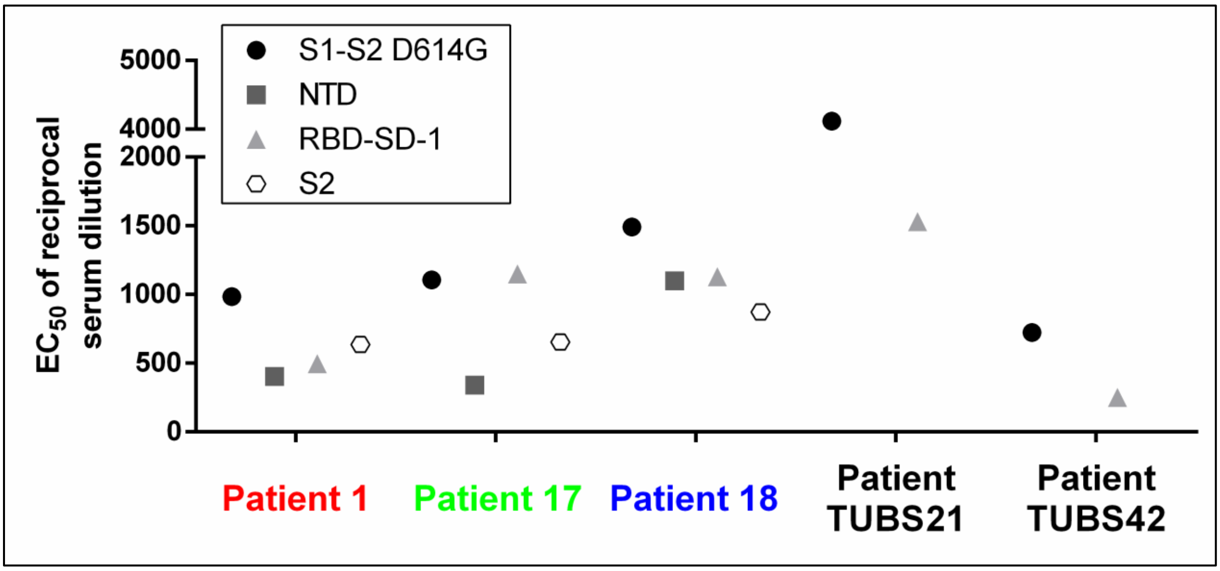

Figure S1: EC_{50} values of reciprocal sera dilutions determined by ELISA.

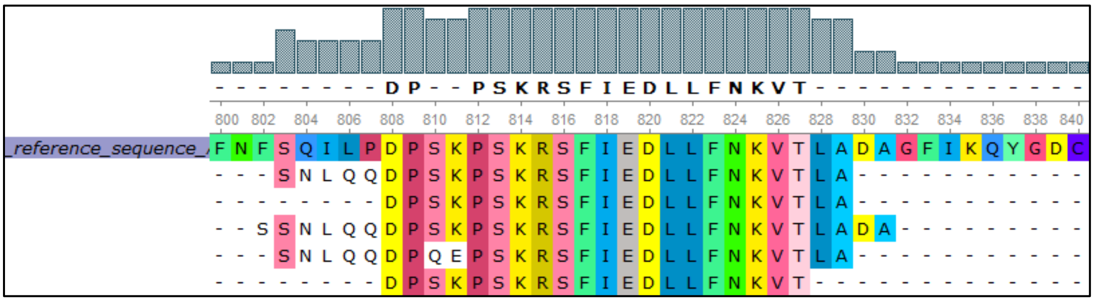

Figure S2: Alignment of selected clones from patient 1 to the Spike protein reference genome (Genbank No.: MT326090.1).

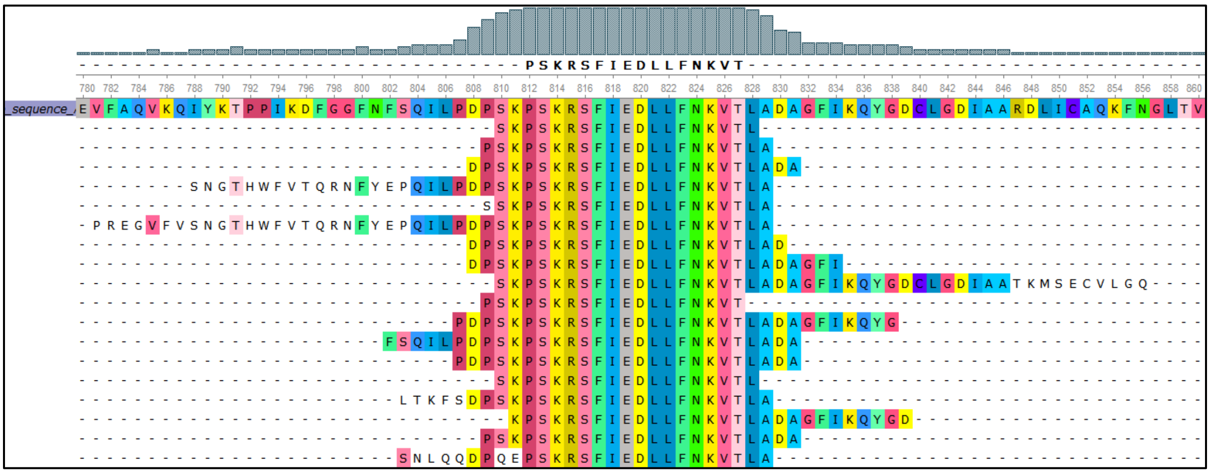

Figure S3: Alignment of selected clones for Spike epitope 1 from patient 18 to the reference genome (Genbank No.: MT326090.1).

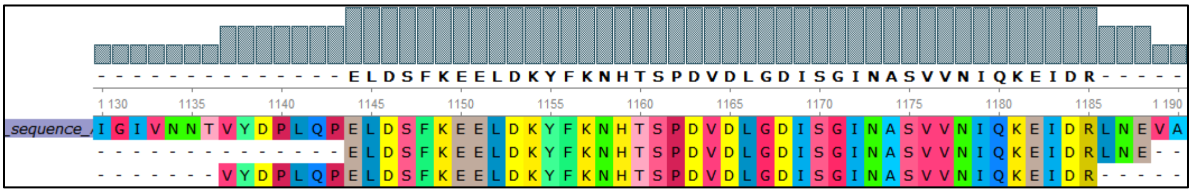

Figure S4: Alignment of selected clones for Spike epitope 2 from patient 18 to the reference genome (Genbank No.: MT326090.1).

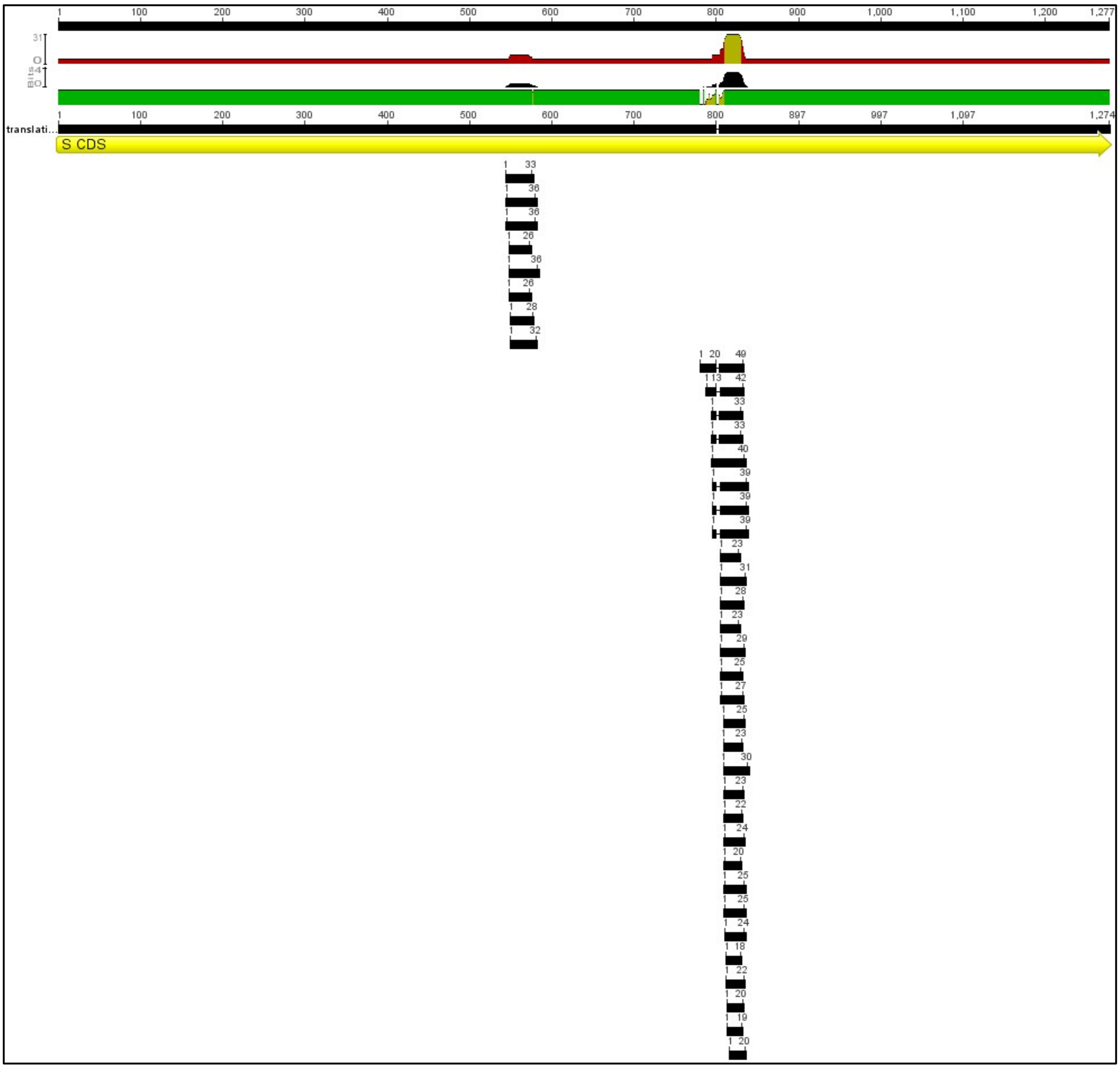

Figure S5: Alignment of all selected clones for both epitopes from patient TUBS21 to the 1274 aa long Spike reference sequence (Genbank No.: MT326090.1).

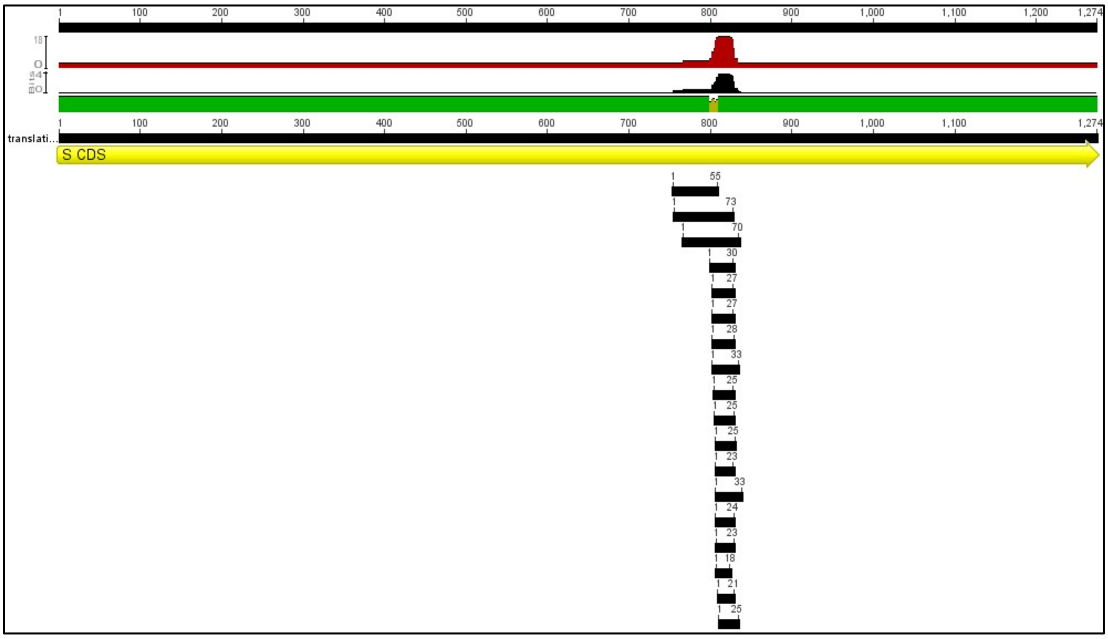

Figure S6: Alignment of all selected clones selected from patient TUBS42 to the 1274 aa long Spike reference sequence (Genbank No.: MT326090.1).
